# Author Correction: A therapeutic combination of two small molecule toxin inhibitors provides broad preclinical efficacy against viper snakebite

**DOI:** 10.1038/s41467-021-24308-0

**Published:** 2021-06-24

**Authors:** Laura-Oana Albulescu, Chunfang Xie, Stuart Ainsworth, Jaffer Alsolaiss, Edouard Crittenden, Charlotte A. Dawson, Rowan Softley, Keirah E. Bartlett, Robert A. Harrison, Jeroen Kool, Nicholas R. Casewell

**Affiliations:** 1 Centre for Snakebite Research and Interventions, Liverpool School of Tropical Medicine, Pembroke Place, L3 5QA Liverpool, UK; 2 Division of BioAnalytical Chemistry, Amsterdam Institute of Molecular and Life Sciences, Vrije Universiteit Amsterdam, De Boelelaan 1085, 1081 HV Amsterdam, The Netherlands; 3 Centre for Analytical Sciences Amsterdam (CASA), 1098 XH Amsterdam, The Netherlands; 4 Centre for Drugs and Diagnostics, Liverpool School of Tropical Medicine, Pembroke Place, L3 5QA Liverpool, UK

**Keywords:** Small molecules, Drug discovery, Diseases

Correction to: *Nature Communications* 10.1038/s41467-020-19981-6, published online 15 December 2020.

The original version of this articles contained an error in Fig. 4. The graphs in panel B report the neutralization of SVSP venom by decreasing concentrations of the inhibitor nafamostat, from 150 μm to 150 nm, but the lowest concentration on the *X-*axis was incorrectly reported as 150 μm. This error has now been corrected on the PDF and HTML version of the article.

