# Supplementary material for: A therapeutic combination of two small molecule toxin inhibitors provides broad preclinical efficacy against viper snakebite
Source: Nat Commun. 2020 Dec 15;11:6094. doi: 10.1038/s41467-020-19981-6 (PMC7738508; doi:10.1038/s41467-020-19981-6)
Supplement: Supplementary file 2 — Reporting Summary [file 41467_2020_19981_MOESM2_ESM.pdf]

nature research

Corresponding author(s): Nicholas Casewell

Last updated by author(s): 28/10/20

Reporting Summary

Nature Research wishes to improve the reproducibility of the work that we publish. This form provides structure for consistency and transparency in reporting. For further information on Nature Research policies, see our Editorial Policies and the Editorial Policy Checklist.

Statistics

For all statistical analyses, confirm that the following items are present in the figure legend, table legend, main text, or Methods section.

n/a	Confirmed	
☐	☒	The exact sample size (n) for each experimental group/condition, given as a discrete number and unit of measurement
☐	☒	A statement on whether measurements were taken from distinct samples or whether the same sample was measured repeatedly
☒	☐	The statistical test(s) used AND whether they are one- or two-sided<br>*Only common tests should be described solely by name; describe more complex techniques in the Methods section.*
☒	☐	A description of all covariates tested
☒	☐	A description of any assumptions or corrections, such as tests of normality and adjustment for multiple comparisons
☐	☒	A full description of the statistical parameters including central tendency (e.g. means) or other basic estimates (e.g. regression coefficient) AND variation (e.g. standard deviation) or associated estimates of uncertainty (e.g. confidence intervals)
☒	☐	For null hypothesis testing, the test statistic (e.g. F, t, r) with confidence intervals, effect sizes, degrees of freedom and P value noted<br>*Give P values as exact values whenever suitable.*
☒	☐	For Bayesian analysis, information on the choice of priors and Markov chain Monte Carlo settings
☒	☐	For hierarchical and complex designs, identification of the appropriate level for tests and full reporting of outcomes
☒	☐	Estimates of effect sizes (e.g. Cohen's d, Pearson's r), indicating how they were calculated

Our web collection on statistics for biologists contains articles on many of the points above.

Software and code

Policy information about availability of computer code

Data collection	FractioMator v1.0 (Spark-Holland), Ariadne v1.8 (VU Amsterdam)
Data analysis	Prism v8 (GraphPad), MARS data analysis v3.31 (BMG Labtech), QGIS v3.10

For manuscripts utilizing custom algorithms or software that are central to the research but not yet described in published literature, software must be made available to editors and reviewers. We strongly encourage code deposition in a community repository (e.g. GitHub). See the Nature Research guidelines for submitting code & software for further information.

Data

Policy information about availability of data

All manuscripts must include a data availability statement. This statement should provide the following information, where applicable:

- Accession codes, unique identifiers, or web links for publicly available datasets
- A list of figures that have associated raw data
- A description of any restrictions on data availability

There are no restrictions on data availability. The raw data supporting the findings of this study and that are displayed in Figures 2, 3, 4, 5, and 6, and Supplementary Figures 2, 4, and 5, can be found in the Supplementary Data File. Data used to construct the species distributions displayed in Fig. 1 are freely available on the World Health Organization Venomous Snake Distribution database (https://apps.who.int/bloodproducts/snakeantivenoms/database/) and the IUCN Red List of Threatened Species database (https://www.iucnredlist.org).

Field-specific reporting

Please select the one below that is the best fit for your research. If you are not sure, read the appropriate sections before making your selection.

- [x] Life sciences
- [ ] Behavioural & social sciences
- [ ] Ecological, evolutionary & environmental sciences

For a reference copy of the document with all sections, see nature.com/documents/nr-reporting-summary-flat.pdf

Life sciences study design

All studies must disclose on these points even when the disclosure is negative.

Sample size	Sample sizes for animal studies were rationally selected based on our prior research (see Albulescu et al. 2020 Science Translational Medicine) and to enable meaningful comparisons between different small molecule toxin therapies (i.e. Supplementary Figure 5).
Data exclusions	Data was not excluded.
Replication	The in vitro assays were performed in triplicate (i.e. three independent experimental runs), with readings for each condition collected in triplicate for each experimental run. The independent experimental runs demonstrated replicability and thus data generated from each experiment was incorporated into downstream analysis.
Randomization	Animal assignation to experimental groups was randomized.
Blinding	Experimenters were blinded to group allocation during animal studies.

Reporting for specific materials, systems and methods

We require information from authors about some types of materials, experimental systems and methods used in many studies. Here, indicate whether each material, system or method listed is relevant to your study. If you are not sure if a list item applies to your research, read the appropriate section before selecting a response.

Materials & experimental systems

n/a	Involved in the study	
x		Antibodies
x		Eukaryotic cell lines
x		Palaeontology and archaeology
	x	Animals and other organisms
x		Human research participants
x		Clinical data
x		Dual use research of concern

Methods

n/a	Involved in the study	
x		ChIP-seq
x		Flow cytometry
x		MRI-based neuroimaging

Animals and other organisms

Policy information about studies involving animals; ARRIVE guidelines recommended for reporting animal research

Laboratory animals	Male CD-1 mice 18-22g (4-5 weeks old)
Wild animals	The study did not involve wild animals.
Field-collected samples	The study did not involve field-collected animals.
Ethics oversight	All animal experiments were performed using approvals from the Animal Welfare and Ethical Review Boards of the Liverpool School of Tropical Medicine and the University of Liverpool and under licensed approval of the UK Home Office (project license #4003718 and #P5846F90).

Note that full information on the approval of the study protocol must also be provided in the manuscript.
